# Preparation and Characterization of Ion-Sensitive Brimonidine Tartrate In Situ Gel for Ocular Delivery

**DOI:** 10.3390/ph16010090

**Published:** 2023-01-08

**Authors:** Haonan Xu, Ye Liu, Lu Jin, Xu Chen, Xinghao Chen, Qiao Wang, Zhan Tang

**Affiliations:** 1 School of Pharmacy, Hangzhou Medical College, Hangzhou 310013, China; 2 Key Laboratory of Neuropsychiatric Drug Research of Zhejiang Province, Hangzhou Medical College, Hangzhou 310013, China

**Keywords:** brimonidine tartrate, in-situ gel, ocular permeation, microdialysis

## Abstract

Brimonidine tartrate (BRT) is a highly selective α2 adrenergic receptor agonist as treatment for patients with open angle glaucoma and high intraocular pressure. The objective of this study was to formulate an ophthalmic ion-sensitive in situ gel (ISG) of BRT to increase the retention time of the drug and its bioavailability. The optimum formulation of 2 mg/mL BRT-ISG was obtained with 0.45% gellan gum as the gel matrix. In vitro release results showed that the water-soluble drug bromonidine tartrate in ocular in situ gels exhibited a high burst effect and fast release in solution. The results of dialysis membrane permeation showed that there was a significant difference between the commercially available and BRT-ISG groups after 45 min. The results of the pre-corneal retention study indicated that gellan gum can effectively prolong ocular surface retention. Preliminary stability results showed that it should be stored in a cool and dark place, and the formulation under long-term preservation can be basically stable. The pharmacokinetic study of the BRT-ISG in the anterior chamber of the rabbit eye was studied by microdialysis technique, and microdialysis samples were analyzed by LC-MS/MS. The pharmacokinetic study showed that the BRT-ISG reached Cmax (8.16 mg/L) at 93 min after administration, which was 2.7 times that of the BRT eye drops, and the AUC(0-t) (1397.08 mg·min/L) was 3.4 times that of the BRT eye drops. The optimal prescription can prolong the retention time of BRT in front of the cornea and significantly improve the bioavailability of BRT in the eye. Combined with the results of in vitro release, permeation and pre-corneal retention studies, the improvement of BRT-ISG bioavailability in rabbit eyes was found to be mainly due to the retention effect after the mixture of ISG and tears.

## 1. Introduction

**Figure 1:**
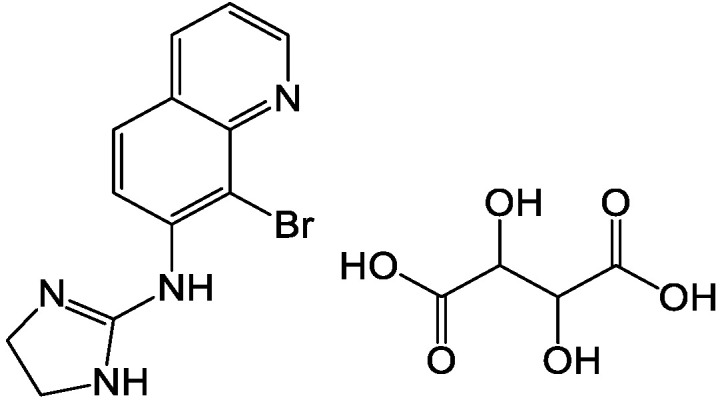
Chemical structure of BRT.

Brimonidine tartrate (BRT, chemical structure shown in Figure 1) is a highly selective α2 adrenergic receptor agonist currently mainly used in clinical practice. It is a promising drug for the treatment of open-angle glaucoma and high intraocular pressure (IOP). Studies using fluorophotometry in animals and humans showed that it has a dual mechanism of IOP-lowering. It can not only reduce the production of aqueous humor, but also increase the scleral-pigmentary outflow of aqueous humor. At the same time, it has an optic neuroprotective effect [1,2]. Local application of a therapeutic dose of BRT has no clinical significance in changes of systolic blood pressure, diastolic blood pressure and heart rate in patients who have been treated for 12 months. Compared with other α receptor agonists, it is relatively less fat-soluble and has difficulty in crossing the blood-brain barrier, which contributes to reducing sedation, hypotension and other central nervous system adverse reactions similar to clonidine. Unlike β-adrenergic receptor antagonists, BRT has a slightly weaker cardiovascular effect and has a weaker effect on lung function [3]. Thus, there is not a contraindication in patients with cardiopulmonary disease. There was no significant difference in the efficacy of BRT and timolol in reducing intraocular pressure [4]. However, timolol-treated low-pressure glaucoma patients were more likely to have field progression than BRT-treated patients. BRT ordinary eye drops are accepted by most patients because of their convenient application and good curative effect. However, due to physiological reasons (such as blinking reflex, tear secretion, nasolacrimal duct drainage, etc.), the BRT is easily removed from the eye surface after administration, resulting in a short stay time in front of the cornea, limited drug absorption, low intraocular bioavailability and difficulties in controlling dosage (frequency of application, ease of overdose). Nasolacrimal duct drainage enables BRT to enter other non-target areas, resulting in side effects [5]. Therefore, it is necessary to develop a new dosage form of the drug.

The above mentioned problems could be overcome by using some bioadhesive materials to increase retention time. Compared to polymer adhesives, in situ gels can avoid discomfort for patients. Compared to thermal and pH-sensitive in-situ gel delivery systems, ion-sensitive in-situ gels have the advantages of low polymer concentration, suitable pH, and no eye irritation [6]. In recent years, some ion sensitive in-situ gel materials have been studied, including gellan gum, carrageenan and sodium alginate [7]. Using acyclovir as a model drug, an ion-activated in-situ gel delivery system based on κ-Carrageenan (KC) has been prepared, which can effectively prolong ocular surface retention time and promote drug penetration [8]. Gellan gum has been used as biomedical material due to its good biocompatibility and low cytotoxicity [9]. By simply mixing with gellan gum solution, a tacrolimus ophthalmic in-situ gel has been developed, which can significantly prolong ocular surface retention time and delay drug release [10]. Sodium alginate and hydroxyl propyl methyl cellulose are combined with azithromycin loaded nano lipid carrier to form in situ gel for eyes. Compared with azithromycin marketed eye drops, the in situ gel system could effectively prolong the drug release time to 24 h and enhance in vitro penetration. The apparent permeability coefficient on the cornea of in vitro goat is 2.61 times higher than that of eye drops [11].

Deacetylated gellan gum is an anionic deacetylated exocellular polysaccharide secreted by Pseudomonas illaceosa, which can be converted into semi-solid gel in the presence of cations [12,13]. Compared with sodium alginate in-situ gel, gellan gum has a faster drug release speed. From the perspective of sustained release, gellan gum may not be the most suitable polymer for sustained release of drugs for eyes [14]. However, studies have compared the effects of K^+^ and Ca^2+^ on the rheological behavior and structural properties of gellan gum, carrageenan and sodium alginate at different concentrations. At 0.5% concentration, carrageenan is mainly affected by K^+^ to undergo solutions-gel phase transition, sodium alginate is mainly affected by Ca^2+^, and gellan gum has a rapid change in rheological behavior except under the influence of Ca^2+^. K^+^ can also affect the rheological behavior of gellan gum. In addition, compared with carrageenan and sodium alginate, gellan gum shows better adhesion and lower cohesion. Considering all indicators, gellan gum is more conducive to the application to eyes [15]. A similar phenomenon was observed in another study evaluating the properties of gellan gum, carrageenan, sodium alginate and their mixtures [16].

For BRT, dendritic macromolecular gels [17], nanoparticles [18] and pH-sensitive in-situ gels [19] and so on have been studied as carriers for intraocular delivery of bromonidine tartrate, all of which have good effects in reducing intraocular pressure. There were biodegradable ophthalmic inserts, but these ocular inserts lack patient compliance [5]. It has also been reported in the literature that the microsphere carrier made of BRT was implanted into rabbit glaucoma conjunctiva [20]. In vivo release studies showed that the release of BRT from microsphere carriers can significantly reduce IOP by 20 mmHg, with a long-term release of 55 days. In addition, carbomer 940 -P was used as a gelling agent, and HPMC K4M was used as a tackifier to prepare BRT pH-sensitive ophthalmic ISG. The polymer used had the strongest gelling ability and had a longer duration of action on the drug, but this dosage form was more irritating to the eyes [19]. Some people in China have also used poloxamer to make 2 mg/mL BRT into a temperature-sensitive ISG which was a mixed matrix of 26% P407 and 1.5% P188; however, the amount of excipients used was too high and would irritate the eyes [21]. Some studies screened the properties of bromonidine tartrate in-situ gel formed based on gellan gum of different concentrations (0.2, 0.4, 0.6, 0.8, 1%), and finally determined the concentration of gellan gum as 0.6%, which had sustained release effect in vitro experiments; there was no pharmacokinetic study [22]. Our pre-experiment results show that when the concentration is higher than 0.5%, the gelling is too strong. First, it is not particularly necessary. Second, it is not conducive to subsequent large-scale production. Generally, the concentration of sustained-release preparations for the eyes is not high, and the whole concentration is stable at a low concentration, which is actually not conducive to the drug taking effect. In addition, the intraocular pressure should be lowered to a certain concentration to take effect, which is similar to the pulse effect. A suitable concentration should be selected which can not only take effect quickly, but also prevent the loss of drugs and the decrease of concentration due to tear flushing. We therefore further screened the concentration of gellan gum.

Microdialysis is a continuous, real-time, in vivo, minimally invasive sampling technique for collecting free compounds in the extracellular fluid of the tissue to be tested based on the principle of dialysis [23,24]. It can be used for long-term continuous sampling on the same live animal, and the sample does not need pretreatment. It can be directly combined with high performance liquid chromatography (HPLC) or liquid chromatography-tandem mass spectrometry (LC-MS/MS) for determination [25]. The animal models of ocular microdialysis mainly include cats, dogs, rabbits and pigeons [26]. The physiological structure of the eyes (size and volume of aqueous humor) of rabbits is most similar to that of humans, and they have further advantages including maneuverability, and being relatively not so expensive; thus, rabbits are commonly used as the ideal model. In order to study the pharmacokinetics of aqueous humor, linear probes are required [27]. But commercial probes are consumable and relatively expensive, and their length and thickness are not the best choice for the pharmacokinetics of the rabbit anterior chamber [28]. Therefore, home-made microdialysis probes were used in pharmacokinetic research of the anterior chamber of BRT rabbit eyes in this research.

In summary, the purpose of this experiment was to prepare and optimize ion responsive BRT-ISG eye drops for ophthalmic delivery, and investigated BRT release in vitro and permeation characteristics, preparation stability and pharmacokinetics of aqueous humor in vivo.

## 2. Materials and Methods

### 2.1. Chemicals

Brimonidine Tartrate Reference Substance (BRT, purity > 98%) was purchased from Hangzhou Deli Chemical Co., Ltd. (Hangzhou, China). Gellan gum was procured from Zhejiang Tianwei Biotechnology Co., Ltd. (TW-JY800, low acyl gellan gum, Zhuji, China). Hydroxypropyl methylcellulose (USP2910, viscosity was 3 mPa·s for 2% Hydroxypropyl methylcellulose) and tromethamine were supplied by Aladdin Reagent Co., Ltd. (Shanghai, China). Mannitol and triethylamine were purchased from Sinopharm Chemical Reagent Co., Ltd. (Shanghai, China). Benzalkonium chloride was provided by Fluka Company. Methanol (Linhai Zhedong Special Reagent Co., Ltd., Linhai, China) and acetonitrile (Tedia^®^, Tedia Company Inc., Fairfield, OH, USA) were HPLC-grade. Sodium chloride, sodium bicarbonate and citric acid were obtained from Chengdu Kelon Chemical Reagent Factory (Chengdu, China). 0.2% brimonidine tartrate eye drops were provided by Allergan, Inc., (Mayo, Ireland). Other reagents and chemicals were of analytical grade.

### 2.2. Animals

New Zealand white rabbits weighing 2.5–3.0 kg of either sex were provided by Hangzhou Yuhang Kelian Rabbit Industry Professional Cooperative, production license number: SCXK (Zhejiang) 2013-0055; Zhejiang Academy of Medical Sciences Laboratory Animal Center license SCXK (Zhejiang) 2014-0008. They were housed in individual cages and maintained on a 12 h light/dark cycle with the room temperature of 24 ± 2 °C in the breeding facility. They were raised following standard protocols of animal care and use guidelines established by the Zhejiang Academy of Medical Sciences, with free access to standard laboratory food and water. All animal experiments were approved by the animal ethics/use committee of Zhejiang Academy of Medical Sciences (ethical approval number: 2019-244).

### 2.3. Formulation Screening

#### 2.3.1. Formulation Preparation

The gellan gum solution was prepared as reported in the literature [29]. The specific formulations composition are shown in Table 1. A certain amount of gellan gum was weighed and dissolved into water under magnetic stirring (water is filtered through a 0.45 μm filter membrane), stirred until the gellan gum had no clumps, and then heated to 90 °C in a water bath to dissolve until it was transparent and completely dissolved. The gellan gum solution was then allowed to cool down at room temperature before making up to volume with water. Preparation of gellan gum/HPMC solution: a certain amount of HPMC was added to 65 °C hot water, and magnetically stirred to disperse it evenly without agglomeration. HPMC solution was left at room temperature for 5 h. The required amount of gellan gum and HPMC solution were evenly mixed, and water was finally added to make the volume up to the mark. Preparation of simulated tear fluid (STF) was according to the electrolyte composition of tears: 0.67% of sodium chloride, 0.20% of sodium bicarbonate, 0.008% of calcium chloride dihydrate [13]. Further, the optimum formulation was screened out based on gelation ability and viscosity. Mannitol was added to adjust osmotic pressure to iso-osmolality, and tromethamine was added to adjust pH to neutrality condition.

#### 2.3.2. Gelation Ability

Gelation ability and viscosity were the two most important factors for ISG formulation screening.

The gelation ability of the gel formulation was to be evaluated to select a composition suitable for ISG. The viscosity was an important factor in determining the retention time of the drug on the ocular surface. Gelation ability measurement: 2 mL STF was taken in a test tube with a stopper in a water bath at 34 °C, then 100 μL of ISG was added to the test tube. The gel formation with or without shaking was observed and recorded [22].

#### 2.3.3. Viscosity Measurement

After the ISG was gelled in STF, it was measured with the No. 52 rotor of the viscometer (DV-2T, Brookfield, Middleborough, MA, USA) [30]. Since the eyes would continuously secrete tears after the actual administration, the blank ISG and the optimal preparation were mixed with different proportions of STF and added to the sample cup. After the samples were equilibrated in the sample cup for 5 min before the test, the viscosity was measured at 10 rpm and 34 °C with different ionic strengths. Then the effect of shear rate on the viscosity of the optimal formulation was investigated under non-physiological conditions (25 °C, ISG:STF = 40:0) and physiological conditions (34 °C, ISG:STF = 40:28), respectively. Each sample was measured 4 times.

### 2.4. In Vitro Release Study

In vitro drug release experiments were carried out via the dialysis bag method [31,32]. Precisely 0.5 mL of 2 mg/mL BRT-ISG preparation or 0.5 mL of 2 mg/mL commercially available BRT eye drops were taken into a dialysis bag (Wuhan Xinsirui Technology Co., Ltd., Wuhan, China, cutoff molecular weight: 14,000), and both ends of the dialysis bag were tied tightly before placing them in 30 mL vials. 20 mL of STF (34 °C) as the diffusion medium were added, and the vials were placed in a thermostatic orbital shaker at 34 °C under a shaking condition of 100 r/min. Samples (1 mL) were taken at intervals of 5, 15, 30, 45, 60, 90, 120 min; isothermal and equal volumes of STF were added immediately. The drug concentration was determined by HPLC. The chromatographic conditions were as follows: chromatographic column, Diamonsil C_18_ column (150 × 4.6 mm, 5 μm); mobile phase, acetonitrile: 0.017 μg/mL citrate buffer (triethylamine was added until the pH = 3) = 0.08:0.92; flow rate of mobile phase, 1.0 mL/min; column temperature, 30 °C; the UV detection wavelength was set at 248 nm; injection volume, 20 µL.

### 2.5. Permeation Studies

#### 2.5.1. Dialysis Membrane Permeation Study

**Figure 2:**
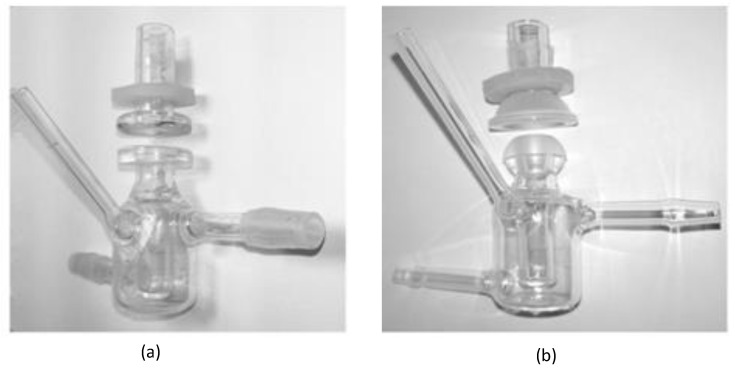
Franz diffusion cell set up for diffusion studies: (**a**) flat-mouth Franz diffusion cell, (**b**) arc-mouth Franz diffusion cell.

In order to better simulate the pre-corneal release behavior of the drug, the in vitro dialysis membrane permeation study was performed by using the dialysis membrane method to compare the BRT-ISG preparation and the commercially available BRT eye drops. The pretreated dialysis membrane (Wuhan Xinsirui Technology Co., Ltd., cutoff molecular weight: 14,000) was embedded between the supply and the receiving pool of the flat-mouth Franz diffusion cell (Figure 2a), and 3 mL of freshly prepared STF solution was added to the receiving pool. The solution was stirred with the magnetic stirrer at 34 °C after the bubbles were removed. Then a mixed solution of BRT-ISG preparation (80 μL) + STF (14 μL) or a mixed solution of commercially available BRT eye drops (80 μL) + STF (14 μL) was added into the supply pools. Samples (100 μL) were taken at pre-determined time intervals (5, 15, 30, 45, 60, 90, 120 min) and isothermal and equal volumes of STF were added immediately. The samples were centrifuged at 9000 rpm for 5 min and the supernatant was determined by HPLC.

#### 2.5.2. Ex Vivo Transcorneal and Transscleral Permeation Studies

Ex vivo transcorneal and transscleral permeation studies were carried out to evaluate the permeation of BRT-ISG and commercially available BRT eye drops in the rabbit cornea and sclera [32,33]. New Zealand white rabbits were sacrificed by intravenous injection of air through the ear vein to induce air emboli. The eyeballs were immediately taken out and the excess tissues were removed. The scleras or corneas were carefully separated. The fresh isolated scleras or corneas were fixed between the supply and the receiving pool of the arc-mouth Franz diffusion cell (Figure 2b) so that the epithelial layer faced the supply pool. Samples were taken at 15, 30, 60, 90, 120, 180, 240 and 300 min timed intervals for transscleral permeation. Samples were taken at 30, 60, 90, 120, 180, 240 300 and 360 min timed intervals for transcorneal permeation. Other operations were the same as in the description of the “Dialysis membrane permeation” method.

#### 2.5.3. Mathematical Modeling of Drug Release Kinetics

The cumulative drug release rate (Q%) is calculated using the following equation:

(1) $$Q_{n}=\frac{V_{0}C_{n}+V\sum_{i=1}^{n-1}C_{i}}{m_{drug}}$$

Q_n_—The cumulative drug release or permeability rate, %;

V—sampling volume, mL;

V_0_—Volume of the medium in the receiving chamber, mL;

C_n_—Drug concentration at sampling points, μg/mL;

C_i_—Drug concentration before sampling points, μg/mL;

m_drug_—Total amount of drug BRT, μg.

The permeability coefficients (Papp) was calculated by formula (Equation (2)) [34].

(2) $$\mathrm{Papp}=(\frac{dC}{dt})\;\times \;V/\left(A\;\times \;C_{0}\right)$$

$\frac{\mathrm{dC}}{\mathrm{dt}}$ is the cumulative drug change in the acceptor concentration calculated from the slope of the time–concentration curve between two time points.

V—buffer volume in the donor compartment (3 mL);

A—diffusional area of the membrane (exposed surface area: 0.53–0.58 cm^2^);

C_0_—initial donor chamber concentration.

The release kinetics studies show the amount of the drug released from the matrix and qualitative changes in formulation designs considered as rational to understand the release mechanism, and generally use the Ritger–Peppas equation. Accoding to the Ritger–Peppas drug release kinetics model, Q = Kt^n^, where “Q” is cumulative release rate, “K” release rate constant, “n” diffusion index and “t” time. When *n* = 1, it means that the release corresponds to zero-order kinetics; *n* = 0.5 means that the release corresponds to the Higuchi equation; *n* < 0.5 means that the in vitro release mechanism is Fick diffusion; 0.5 < *n* < 1 means the in vitro release mechanism is a combination of erosion and diffusion [35,36].

### 2.6. Pre-Corneal Retention Study

The experiment was carried out by the fluorescein sodium labeling method [37,38]. The specific method was to mix 1% sodium fluorescein aqueous solution and gellan glue solution (0.5%) in proportion (1:9) to obtain 0.1% sodium fluorescein gellan gel solution (Fls-gel), and prepare 0.1% sodium fluorescein aqueous solution (Fls-solu). 50 μL Fls-gel and Fls-solu were added to the conjunctival sac of healthy rabbits, and the eyes of rabbits were closed for 10 s. The changes of green fluorescence with time were observed under cobalt blue light. Images were collected before administration (Control) and 1, 3, 5, 10, 15, 20, 25, 30, 40, 50 and 60 min after instillation. The experiment was conducted in a dark room.

### 2.7. Ocular Irritation Studies

The test was performed according to the modified Draize test in order to evaluate the toxicity of the BRT-ISG [39]. Both left and right eyes of the rabbits were used to self-contrast, and one eye was instilled with BRT-ISG, while the other eye was instilled with commercially available BRT eye drops as a control. During the experiment, the head of the animal was tilted to the right so that the left eye of the animal was tilted upward, and the lower eyelid of one eye is gently lifted. Eye drops should be instilled directly in the conjunctiva sac and the eyelid should be closed gently for 10 s after putting 1 drop. As for the right eye, dripping therapy was applied in the same manner for 14 days continuously. Ocular stimulation response was examined and scored visually and with a magnifying glass before the first dose each day as well as 1, 2, 4, 24, 48, and 72 h after the last dose.

### 2.8. Stability Studies of BRT-ISG

The BRT-ISG contents were determined using HPLC by the method described in the section above, “In vitro release study”. The chromatographic conditions of impurities concentration determined by HPLC were as follows: chromatographic column, Diamonsil C_18_ column(150 × 4.6 mm, 5 μm); mobile phase, acetonitrile (A) and 0.017 μg/mL citrate buffer (triethylamine was added until the pH = 3) (B)—gradient eluted was programmed as follows: 0.01–1.00 min 8% eluent A, 1.01–5.00 min 8–20% eluent A, 5.01–13 min 20% eluent A, 13.01–22.00 min 8% eluent A; flow rate of mobile phase, 1.0 mL/min; column temperature, 30 °C; the UV detection wavelength was set as follows: 0.01–6.50 min 248 nm, 6.51–16.00 min 266 nm, 16.01–22.00 min 248 nm; injection volume, 20 µL.

BRT-ISG stability tests were evaluated using an influencing factor experiment including high temperature and strong light (Chinese Pharmacopoeia 2020 (fourth part, Guidelines for stability testing of active pharmaceutical ingredient and preparations). The preparations were divided among light blue high-density polyester eye drop bottles (Suzhou Jinxin Medical Plastic Container Factory), sealed and kept for samples. The samples were placed in a constant temperature oven at 40 °C and 60 °C for 10 days. Samples were taken on the 5th and 10th days to investigate various indicators of the preparations. The samples were placed in a light incubator equipped with a fluorescent lamp under the condition of 4500 lx ± 500 lx for 10 d, and samples were taken on the 5th and 10th days to investigate the various indicators of the samples.

The accelerated stability tests were conducted at a temperature of 40 ± 2 °C and relative humidity of 25 ± 5% (saturated potassium acetate solution) for 6 months. Samples were taken at 1st, 2nd, 3rd, and 6th months to investigate the various indicators of the samples.

### 2.9. Study on the Pharmacokinetics of Ophthalmic BRT-ISG in Rabbit Anterior Chamber

The chromatographic conditions were as follows: chromatographic column ZORBAX SB-C18 (3.0 × 150 mm, 3.5 μm); mobile phase, 0.1% formic acid water (A), acetonitrile (B), isocratic elution program: A:B = 50:50; flow rate, 0.4 mL/min; injection volume, 5 μL; column temperature, 40 °C. The MS conditions were as follows: positive (ESI+) electrospray ionization mode: multiple reaction monitoring (MRM) mode; ion source parameters, gas temperature 300 °C, gas flow 5 L/min, nebulizer 45 psi, sheath gas temperature 250 °C, sheath gas flow rate 11 L/min, capillary voltage 3500 V, nozzle voltage 500 V. The detailed method of HPLC-MS/MS analysis of BRT can be seen in our previous publication [40]. On the basis of this analytical method, we have chosen to increase sample size and animal experiments, in order to further investigate the pharmacokinetics in aqueous humor of BRT-ISG. Detailed information can be found below.

The in vivo pharmacokinetics study was performed by employing the microdialysis technique which was modified slightly from the previous literature [27,41]. Simply, New Zealand white rabbits were kept anesthetized throughout the experiment by intravenous injection with 25% urethane (1 g/kg) from ear vein. Then the peripheral skin around the eyes was exposed, and the rabbits were subsequently placed in the prone position with their limbs and head fixed on a thermostatic pad of the operating table at a temperature of 37 °C. The pupils were dilated with tropicamide eye drops before operation. The rabbits eyelids were kept open using suitable eyelid opener, then a 25 G injection needle was inserted carefully into the anterior chamber by piercing in from one edge of the cornea and out at the other edge. The microdialysis probe (membrance length 7.5 mm) was implanted into the anterior chamber via the lumen of the needle, the needle was removed, leaving the membrane of the probe in the anterior chamber of the rabbit completely. The puncture wound at the implant sites was repaired with tissue glue. Before administration, the probe was perfused with blank Ringer’s solution by a microinjection pump (MD-1001) for 2 h at a flow rate of 1.0 μL/min. After the steady state of eyes was reached, the rabbits were given 50 μL of BRT-ISG eye drops or commercially available BRT eye drops (BRT ≈ 0.1 mg). Then the microdialysis samples were collected every 30 min (30 µL of each aqueous humor sample) within 360 min after administration and analyzed by LC-MS/MS directly without further treatment. The actual concentration of BRT in the aqueous humor (Cr) was determined as the ratio of the measured concentration of microdialysis fluid (C_m_) to the recovery rate (R_dial_), C_r_ = C_m_/R_dial_ [28]. The sample was collected continuously each 30 min, and the calculation result was the average concentration of each sampling time period; therefore, the midpoint of time period was used as abscissa to plot the drug concentration-time curve.

## 3. Results and Discussion

### 3.1. Formulation Screening

#### 3.1.1. Gelation Ability

The gelling ability of each formulation was investigated, and the results are shown in Table 1. It was found that when the concentration of gellan gum was above 0.6%, it was relatively viscous at room temperature. While when the concentration of gellan gum was 0.3% and below, the gelling ability was relatively poor. Therefore, the 0.5%, 0.45%, 0.4% concentrations of gellan gum were selected for formulation screening. According to literature reports, the combination of gellan gum and the tackifier HPMC can increase the strength of the gel [14,42]. It was found that when the gellan gum concentration was in the range of 0.4–0.5%, or/and in combination with HPMC, it could quickly form a gel after mixing with STF and would not easily disperse after shaking.

#### 3.1.2. Viscosity Measurement

**Figure 3:**
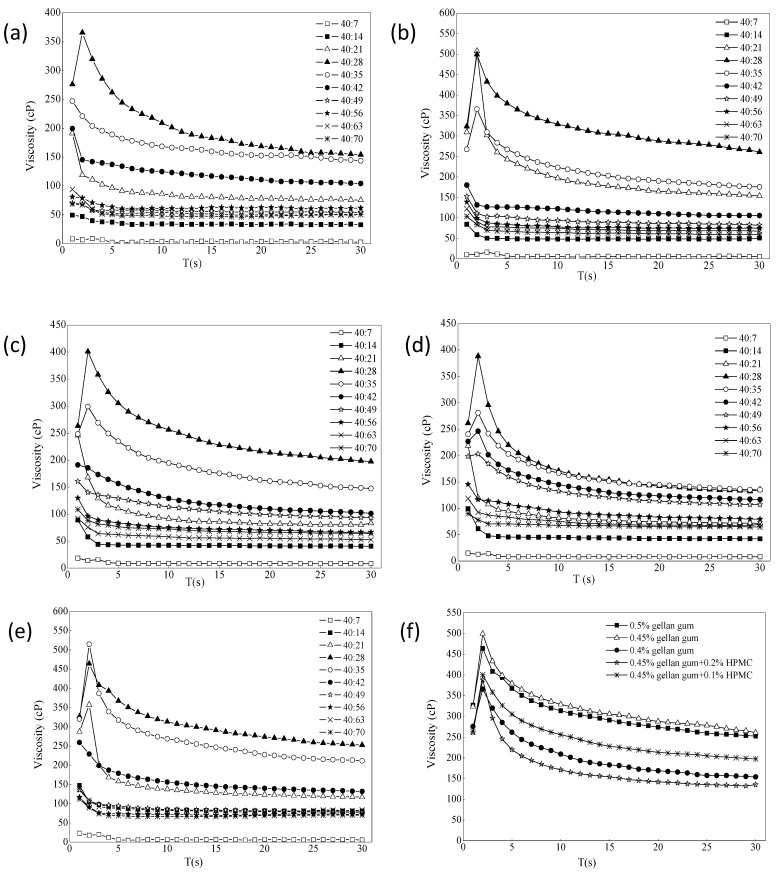
(**a**–**e**) The effect of ion intensity on the viscosity of different formulations ((**a**) 0.4% gellan gum; (**b**) 0.45% gellan gum; (**c**) 0.45% gellan gum and 0.1% HPMC; (**d**) 0.45% gellan gum and 0.2% HPMC; (**e**) 0.5% gellan gum). (**f**) The value of viscosity change of the formulations STF = 40:28 (34 °C, 10 rpm, *n* = 4).

The effect of ion intensity on the viscosity of different formulations is shown in Figure 3a–e. It usually increased in the first few laps, and the resistance of the liquid was not felt at the beginning. When the resistance was felt completely, it would increase slowly. When it decreased later, most of the samples were pseudoplastic, and it would decrease slowly as the shear became thinner. The different ratios of gel with STF from 40:7 to 40:70 were chosen because the instilled volume of eye drops is about 40 μL and the normal tear fluid volume is 7 μL. The renewal rate of the lachrymal fluid is 1.2 μL per minute [43,44]. The purpose of adding different portions of STF is to simulate the change of viscosity of the preparations during the continuous secretion of tear fluid. It can be seen that the viscosity of each formulation increased first and then decreased with the addition of STF. When it was added to a certain ratio, the viscosity tended to be flat. The viscosity value was the largest when ISG:STF ratio was 40:28, and formulation 3 could hold more STF. As shown from Figure 3f, comparing the viscosity values of each formulation when the STF ratio was 40:28, the viscosity changes of formulation 3 and formulation 6 were the largest, indicating that both formulations had stronger resistance to tear dilution. However, the viscosity of formulation 6 under non-physiological conditions (18.0 cP) was twice that of formulation 3 (8.8 cP), and the liquidity was less than formulation 3. After gellan gum was combined with HPMC, the viscosity change value did not increase significantly, which may be due to the different types of excipients and different measurement conditions from those reported in the literature [14,42]. Therefore, according to the principle of fewer excipients, gellan gum alone was selected as the optimal gel matrix in this study.

**Figure 4:**
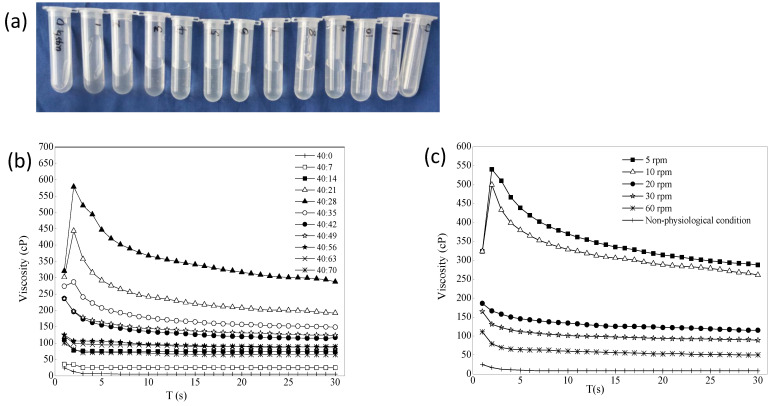
(**a**) 0.45% Gellan gum mixed with artificial tears in different ratios (from left to right: 0.45% gellan gum/STF from 40:0, 40:7, 40:14 to 40:84). (**b**) The effect of ion intensity on the viscosity of the optimal BRT-ISG (34 °C, 10 rpm, *n* = 4). (**c**) The effects of shear rate on the viscosity of the 0.45% gellan gum:STF = 40:28 (non-physiological was 25 °C, the other was 34 °C, *n* = 4).

Figure 4a is a schematic diagram of a mixture of 0.45% gellan gum/STF from 40:0 to 40:84. A total of 13 mixture samples were laid flat in a row after 20 min at room temperature. The blank gellan gum had good fluidity. The mixture of 0.45% gellan gum:STF ratio of 40:7 showed that the gelling ability was not strong, and the result of the viscosity value was also small. This may be because the cation concentration was too low, and gellan gum did not fully form a high molecular polymer gel with a network structure. When the gellan gum:STF ratio was less than 40:70, the gelling ability did not increase further but weakened, and the mixed liquid showed a flowing state. 0.2% BRT was added to formulation 3 as the optimal formulation, which contained mannitol (4.2%, to adjust the osmotic pressure to isotonic), tromethamine (0.06%, to adjust the pH) and benzalkonium chloride (0.01%, to use as the bacteriostatic agent). The selected optimal formulation had an osmolality molar concentration of 286 mOsmol/kg measured with STY-1A osmometer (Tianda Tianfa Technology Co., Ltd., Tianjin, China), an osmotic pressure ratio of 1.0, and a pH of 6.70 (pH meter PHS-3C). Figure 4b shows that the viscosity of the optimal BRT-ISG formulation was mixed with different proportions of STF, and the resulting trend was the same as that when the blank ISG was mixed with STF. The effects of shear rate on the viscosity of formulation 3 were examined. Because the viscosity change value was the largest when the blank ISG:STF ratio was 40:28, this ratio was chosen to investigate the influence of the shear rate on the ISG, as shown in Figure 4c. The experiment was repeated four times for each sample. Mean ± standard deviation (SD) are not shown because Figures cannot display such small differences. The viscosity of the non-physiological condition (25 °C, ISG:STF = 40:0) was very low, and the viscosity of the gel increased when it was changed to the physiological condition (34 °C, ISG:STF = 40:28). In addition, the viscosity of the ISG decreased as the shear rate increased, showing non-Newtonian fluid characteristics and shear thinning characteristics.

### 3.2. In Vitro Release Study

**Figure 5:**
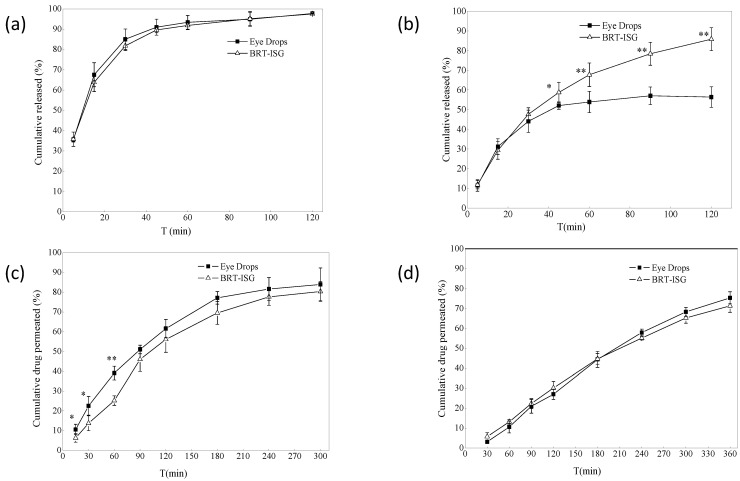
(**a**) In vitro release profiles of dialysis bag model (*n* = 4, $\bar{x}$ ± s). (**b**) Dialysis membrane permeation profile (*n* = 6, $\bar{x}$ ± s). * *p* < 0.05 BRT-ISG vs. eye drops at 45 min, ** *p* < 0.01 BRT-ISG vs. Eye Drops at 60, 90, 120 min. (**c**) Ex vivo transscleral permeation profile (*n* = 5, $\bar{x}$ ± s). * *p* < 0.05 BRT-ISG vs. eye drops at 15, 30 min, ** *p* < 0.01 BRT-ISG vs. eye drops at 60 min. (**d**) Ex vivo transcorneal permeation profile (*n* = 5, $\bar{x}$ ± s).

The dialysis bag method was used for the formulations’ in vitro drug release study, and the commercially available eye drops were used as the control group. The in vitro release results of the dialysis bag are shown in Figure 5a. The self-prepared BRT-ISG group released a cumulative release of 93% at 60 min, the commercial group released a cumulative release of 91%, and both released 97% at 120 min. There was no significant difference between the two groups (*p* > 0.05). The results showed that both the BRT-ISG group and commercial group gave a rapid release of the BRT, which could take effect quickly after intraocularly delivered. Origin8.0 software was used to fit the data of the self-prepared BRT-ISG cumulative release rate in the range of 0–120 min of mathematical models. The results are shown in Table 2. The results showed that only the first-order kinetic equation could fit the self-prepared data well. Therefore, the drug release mechanism when measured by the dialysis bag method was Fick diffusion. In some studies, after a water-soluble drug was prepared in an in situ gel, it still had a sudden release phenomenon like an aqueous solution [32]. Such drug release behavior maybe determined by the physical and chemical properties of the drug. Therefore, in this study, there is no difference in the drug release between the BRT-ISG and the commercially available eye drops, which may be due to the sudden release effect.

### 3.3. Permeation Studies

#### 3.3.1. Dialysis Membrane Permeation Study

The dialysis membrane was also used to study the formulations in vitro using a flat-mouth Franz diffusion cell, with the commercially available eye drops as a control. The results for self-prepared BRT-ISG are shown in Figure 5b. There was a significant difference between the two groups at 45 min (*p* < 0.05), and very significant difference after 60 min (*p* < 0.01). The self-prepared BRT-ISG had a cumulative release rate of 85% at 120 min, while the commercial group has a cumulative release rate of 52% at 45 min, which tends to be stable, indicating that the self-prepared BRT-ISG had retention characteristics on the flat-mouth Franz diffusion cell. The reason for this phenomenon may be that BRT-ISG was concentrated in the middle part of the flat-mouth Franz diffusion cell due to its viscosity while the position of the eye drop solution group could easily lead to loss from the mouth of the Franz diffusion cell. The self-prepared BRT-ISG cumulative release rate was fitted with mathematical models, and the results are shown in Table 2. According to the results, except for the zero-order kinetic equation, the other three equations can better fit the BRT-ISG drug release process. Since gellan gum is a hydrophilic gel, the first-order kinetic equation and Higuchi equation can only roughly simulate its release behavior. According to the fitting result of the Ritger–Peppas equation, *n* = 0.564. Since 0.5 < *n* <1, this showed that the in vitro release mechanism of the preparation dialysis membrane method was combined erosion with diffusion.

#### 3.3.2. Ex Vivo Transcorneal and Transscleral Permeation Studies

The ex vivo permeation study of formulations were carried out using the isolated sclera, and the commercially available eye drops were used as a control. The ex vivo permeation results of self-prepared BRT-ISG are shown in Figure 5c. Before 90 min, the two groups had significant differences (*p* < 0.05). The cumulative permeability of the self-prepared BRT-ISG was significantly lower than that of the commercial group, indicating that the self-prepared BRT-ISG had a certain slow-release effect, and the cumulative permeability of 300 min was more than 80%. The sclera cumulative permeability of the self-prepared BRT-ISG sclera was fitted by mathematical models and the results are shown in Table 2. The release mechanism was combined erosion with diffusion.

Drugs were absorbed through the eye and generally needed to pass through ocular tissues such as cornea, sclera and conjunctiva. Therefore, the ex vivo corneal permeability was investigated using cornea. The results are shown in Figure 5d. The 6-h cumulative permeability of the self-prepared BRT-ISG was 71%, and the cumulative permeability of the commercially available group was 75%. There was no significant difference between the two groups using the paired Students T-test analysis (*p* > 0.05). Mathematical models were applied to fit the results of the self-prepared BRT-ISG corneal cumulative permeability as shown in Table 2. The drug release mechanism was combined erosion with diffusion. The permeability coefficient of eye drops in the sclera was 35.91 × 10^−3^ (cm/h), and that of in situ gel was 36.24 × 10^−3^ (cm/h). The permeability coefficient of eye drops in the cornea was 32.11 × 10^−3^ (cm/h), and that of in situ gel was 35.36 × 10^−3^ (cm/h). There was no significant difference in the permeability efficiency of the two preparations on the sclera and cornea calculated by software GraphPad Prism 8.0. A pH responsive in-situ gel prepared with carbomer was reported [19]. Its corneal permeability in 5 h was 76.83%, and the permeability coefficient was 28.25 × 10^−3^ (cm/h). In another study, a scholar used polyamidoamine (PAMAM) to prepare an ophthalmic polymer hydrogel to realize the co-delivery of bromonidine tartrate and timolol maleate [17]. Bromonidine tartrate achieved 74% corneal penetration in 6 h, and the permeability coefficient was 7.6 × 10^−5^ cm/s. The permeability coefficient is 35.36 × 10^−3^ (cm/h) of the in-situ gel in this study, equivalent to 0.98 × 10^−6^ cm/s. Compared with the other two preparations, the permeation efficiency is similar. The permeability coefficient of the in-situ gel in this study is higher than that of pH responsive in-situ gel and lower than that of polymer gel. To a certain extent, the in-situ gel of this study achieved the effect of sustained release. However, ionic in-situ gel is more convenient for storage and transportation than polymer gel. Compared with pH responsive in-situ gel, it is less affected by the environment, and the ions in tears can complete the phase transition. The cumulative drug permeation ex vivo was similar for the two preparations (commercially eye drops and BRT-ISG groups) on the sclera and cornea, because the permeation cell was an upper and lower arc structure, the cornea and sclera were stretched on the cell mouth, and the upper and lower cell mouths fit tightly; there was no fluid loss of commercially available eye drop from cell, so there was more permeation, which cannot reflect the better retention effect of ISG than solution. Therefore, in vivo pharmacokinetic experiments were needed for further verification.

It has been reported that gellan gum was used as the gel matrix of BRT, with the best concentration of 0.6%, which had a sustained-release effect in an in vitro experiment only studying cellophane membrane using a Franz diffusion apparatus [22]. In our experiment, the optimal concentration of gellan gum was 0.45%, and the release study (dialysis bag method) and the permeation study (dialysis membrane, transcorneal and transscleral) were all carried out, which showed the prescription had the dual characteristics of rapid release and continuous release. FDA stipulates that the safe ocular use concentration of gellan glue is within 0.6%. If this maximum limit is used, first, gelling is too firm and releases slowly, which is not suitable for rapid onset. In addition, the viscosity of the preparation at room temperature is too high and the fluidity is poor, which is not suitable for industrial filling and mass production.

### 3.4. Pre-Corneal Retention Study

**Figure 6:**
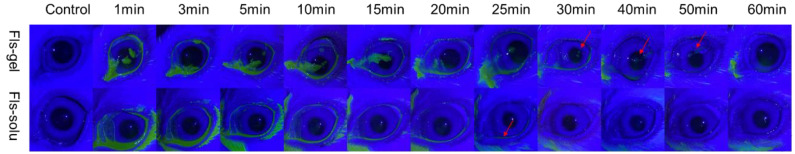
In vivo fluorescence imaging of Fls-gel and Fls-solu at various time points after instillation.

The change of ocular surface fluorescence with time after the instillation of Fls-gel and Fls-solu is shown in Figure 6. In the Fls-solu group, the green fluorescence was significantly reduced after 5 min and basically disappeared after 25 min (as shown by the red arrow), while in the Fls-gel group, there was still obvious green fluorescence in the ocular surface at 25 min. The fluorescence was weak at 50 min and disappeared completely at 60 min. These results indicated that gellan gum can effectively prolong ocular surface retention. Some relevant studies have been reported in recent years, in situ gel for curcumin eye drops was prepared with gellan gum (the concentration of gellan gum was 0.5%), labeled with fluorescein sodium (0.1%) and then dropped on the rabbit eye surface. Under cobalt blue light, the green fluorescence disappeared at 50 min, while the fluorescein sodium solution significantly decreased at 5 min, which was similar to the results we observed [45].

### 3.5. Ocular Irritation Studies

**Figure 7:**
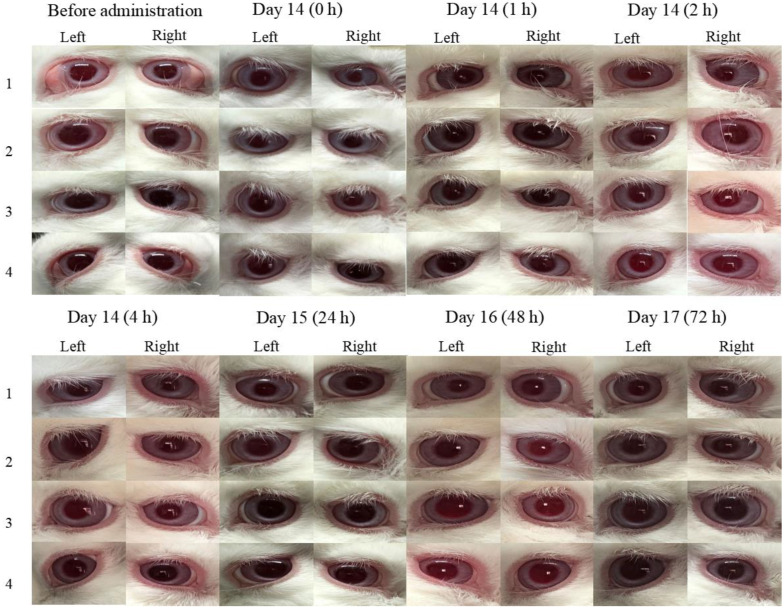
The obtained data of at 0, 1, 2, 4, 24, 48, and 72 h after the instillation of BRT-ISG and commercial product of BRT 14d continuously.

Figure 7 demonstrates that the obtained data of ocular irritation experiments at 0, 1, 2, 4, 24, 48, and 72 h after the instillation of BRT-ISG and commercial product of BRT 14d continuously. As measured by the ocular irritation score scale, both preparations showed no corneal opacity, normal sclera, normal conjunctival vascular system, no edema, as well as no discharge. This means that all five aspects of cornea, sclera, conjunctiva, edema, and secretion score 0 (out of 16). Overall, the evaluation indicates that there was no irritation of the eyes.

### 3.6. Stability Studies of BRT-ISG

The results of the high temperature test and intense light test are shown in Table 3. The results showed that compared with the initial condition, after standing at high temperature of 40 °C and 60 °C for 10 days, the appearance, pH value, content and impurity content were basically unchanged. The osmotic pressure was basically unchanged at 40 °C, while the osmolality increased slightly at high temperature of 60 °C, but it was still within the range of 260–310 mOsmol/kg which meeting the requirements of human eyes.

The results of intense light test showed that compared with the initial condition, after being placed for 10 days under the condition of 4500 lx ± 500 lx, the appearance, pH value and content of the preparation were basically unchanged, but the impurity content increased, indicating that the BRT preparation should be stored in the dark.

The results of the accelerated test are shown in Table 4. Compared with the initial period, after 6 months of accelerated storage, the appearance and content were basically unchanged, and the pH value, osmotic pressure and impurity content increased slightly, but the total impurities were controlled below 0.5%, single impurity was below 0.2%. It is expected that the formulations will be stable after long-term storage.

1-bromoquinoxalinyl-6-thiourea was selected as the most representative related impurity for detection [46,47]. The stability tests investigated the appearance, pH value, osmotic pressure and other aspects, and provided a basis for future drug preparation process screening, packaging materials, and storage conditions. It had been reported in the literature that BRT preparations were affected by strong light during storage. The results of this study showed that although the impurity content was slightly increased, it was still within the limit; it is best to store it in the dark [10]. Storage at a high temperature of 60 °C had a certain effect on the preparation, and the osmotic pressure increased slightly, indicating that the preparations should be stored in a cool place. The formulation was basically stable within 6 months under accelerated conditions of 40 °C. Although the pH, osmotic pressure and impurity content increased slightly, the BRT-ISG formulations were generally stable, and the period of validity can be tentatively set at 2 years.

### 3.7. Study on the Pharmacokinetics of Ophthalmic BRT-ISG in Rabbit Anterior Chamber

**Figure 8:**
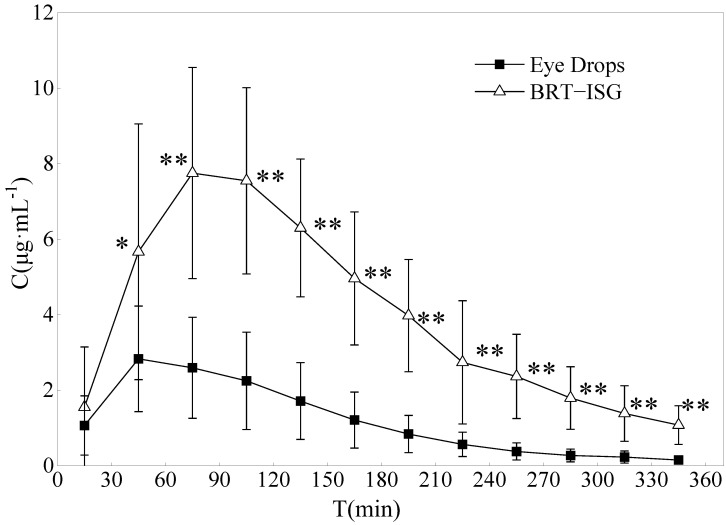
Concentration of Brimonidine in the aqueous humor of rabbits after administration of eye drops and BRT−ISG (*n* = 10, $\bar{x}$ ± s). * *p* < 0.05 and ** *p* < 0.01 BRT−ISG vs. eye drops.

The drug concentration-time curves of the two preparations in the rabbit aqueous humor are shown in Figure 8. The DAS2.0 analysis software was used for analysis, and the main pharmacokinetic parameters were obtained by non-compartmental model (statistical moment), as shown in Table 5. The commercial group reached C_max_ (3.06 mg/L) about 60 min after administration, while the self-prepared BRT-ISG group reached C_max_ (8.16 mg/L) about 93 min after administration. The results showed that the C_max_ of BRT-ISG was significantly higher than that of the commercially available eye drops. The MRT_(0-t)_ of self-prepared BRT-ISG group was 1.3 times later than the commercial group, indicating that as the retention time of the drug in front of the cornea was prolonged, the drug could continue to penetrate the cornea into the aqueous humor. The AUC_(0-t)_ of the self-prepared BRT-ISG group was 1397.08 ± 444.57 mg·min/L, which was 3.4 times higher than that of the commercial group. The two groups had extremely significant differences (*p* < 0.01). The bioavailability of BRT-ISG eye drops group in the eye was significantly improved.

Microdialysis sampling has been used in pharmacokinetic studies of BRT in aqueous humor after topical administration of commercially available BRT solution. It was a well known traditional sampling method requiring sacrificing a large number of animals. The sample pretreatment procedure, which involved solid-phase extraction, was tedious and subject to error and time-consuming. According to literature reports on the pharmacokinetics of aqueous humor after local instillation of Alphagan^®^ eye drops, its concentration in aqueous humor was lower than the results of this research; these differences could be due to different administered doses [48,49]. Rabbits were given a single 35 μL drop of Alphagan^®^ (0.2% brimonidine tartrate) in the eyes in their experiment, while 50 μL drops were given in this study. Mathematically, the results of AUC and C_max_ would be close to our study if dose influence were removed. This meant that, the results obtained by the two different sampling methods were consistent. Combined with the results of in vitro release and permeation experiments, the improvement of BRT-ISG bioavailability in rabbit eyes was mainly due to the retention effect after the mixture of ISG and tears.

## 4. Conclusions

The current commercially available BRT eye drops have the disadvantages of fast loss, short effective time, and poor safety. In this study, an ion-sensitive ophthalmic BRT in situ gel was optimized. This preparation is simple and convenient to prepare, has good fluidity at room temperature, is suitable for industrial production, and has good stability. BRT-ISG can be released quickly, which means it can take effect quickly after intraocular administration. In vivo experiments provided evidence that the BRT-ISG formulation can prolong the retention time in the eye and significantly improve the bioavailability of BRT. The optimal prescription had the dual characteristics of rapid release and continuous release. In the future, it is hoped that the new BRT-ISG preparation can be developed into a new drug to market, providing safer, more effective and convenient dosage forms for the majority of patients. It has the characteristics of rapid release and continuous release, and its intraocular bioavailability is significantly higher than that of commercial ordinary eye drops.

## Figures and Tables

**Table 1:** The gelling capacity of the formulations.

Formulations	Gellan Gum/%	HPMC/%	Gelling Capacity
1	0.3		+
2	0.4		++
3	0.45		++
4	0.45	0.1	++
5	0.45	0.2	++
6	0.5		++
7	0.6		++

Note: (+) immediate gelation and disperse easily; (++) immediate gelation, swaying was not easy to disperse.

**Table 2:** Model fitting and parameters of in vitro drug release and permeation studies.

Methods	Zero-Order Equation	First-Order Equation	Higuchi Equation	Ritger–Peppas Equation	
	R^2^	R^2^	R^2^	R^2^	*n*
Dialysis bag model	0.548	0. 963	0.680	0.804	0.157
Dialysis membrane permeation model	0.862	0.997	0.980	0.968	0.564
Transscleral permeation model	0.932	0.984	0.978	0.963	0.754
Transcorneal permeation model	0.978	0.985	0.992	0.980	0.950

**Table 3:** Stability of BRT-ISG under 40 °C, 60 °C and high light (*n* = 3).

Temperature /°C	Time /Day	Appearance Characters	pH	Osmolarity /mOsmol/kg	Contents /%	Impurity Contents/%
	0	light yellow, odorless and transparent	6.74 ± 0.05	289.0 ± 1.0	99.96 ± 0.25	Signal 0.0% All 0.0%
40	5	light yellow, odorless and transparent	6.72 ± 0.04	288.0 ± 2.6	100.51 ± 0.03	
	10	light yellow, odorless and transparent	6.71 ± 0.03	292.0 ± 4.2	101.83 ± 0.52	Signal 0.0% All 0.04%
60	5	light yellow, odorless and transparent	6.75 ± 0.03	296.0 ± 2.6	102.22 ± 0.05	
	10	light yellow, odorless and transparent	6.79 ± 0.02	302.0 ± 4.0	100.67 ± 0.35	Signal 0.0% All 0.02%
4500 lx ± 500 lx	5	light yellow, odorless and transparent	6.73 ± 0.05	288.0 ± 1.5	99.40 ± 0.05	
	10	light yellow, odorless and transparent	6.75 ± 0.03	292.0 ± 3.1	100.86 ± 0.22	Signal 0.02% All 0.41%

**Table 4:** Accelerated stability of BR-ISG (*n* = 3).

Temperature /°C	Time /Month	Appearance Characters	pH	Osmolarity /mOsmol/kg	Contents /%	Impurity Contents/%
	0	light yellow, odorless and transparent	6.74 ± 0.05	289.0 ± 1.0	99.96 ± 0.25	Signal 0.0% All 0.0%
40	1	light yellow, odorless and transparent	6.73 ± 0.02	296.0 ± 2.1	99.24 ± 0.86	Signal 0.02% All 0.06%
	2	light yellow, odorless and transparent	6.75 ± 0.03	294.0 ± 1.5	99.11 ± 1.02	Signal 0.05% All 0.14%
	3	light yellow, odorless and transparent	6.79 ± 0.02	298.0 ± 2.5	100.46 ± 1.02	Signal 0.06% All 0.15%
	6	light yellow, odorless and transparent	6.85 ± 0.04	301.0 ± 2.9	100.55 ± 0.19	Signal 0.14% All 0.33%

**Table 5:** The main pharmacokinetic parameters of Brimonidine in aqueous humor after topical administration (*n* = 10, $\bar{x}$ ± s).

Parameter		Eye Drops	BRT−ISG
AUC_(0-t)_	mg·min/L	412.61 ± 204.31	1397.08 ± 444.57 **
AUC_(0-∞)_	mg·min/L	426.34 ± 212.54	1514.91 ± 504.58 **
MRT_(0-t)_	min	110.28 ± 18.17	140.06 ± 17.50 **
MRT_(0-∞)_	min	121.04 ± 20.02	165.31 ± 22.36 **
t_1/2_	min	64.99 ± 19.18	75.60 ± 17.66
T_max_	min	60 ± 21.21	93.00 ± 28.98 **
C_max_	mg/L	3.06 ± 1.42	8.16 ± 2.62 **

** *p* < 0.01 BRT-ISG vs. eye drops.

## Data Availability

All experimental data relevant to this study are contained within the article.
